# Thermally Induced Knudsen Forces for Contactless Manipulation of a Micro-Object

**DOI:** 10.3390/mi13071092

**Published:** 2022-07-10

**Authors:** Clint John Cortes Otic, Shigeru Yonemura

**Affiliations:** 1 Department of Finemechanics, Graduate School of Engineering, Tohoku University, 6-6 Aramaki Aza Aoba, Aoba-ku, Sendai 980-8579, Japan; 2 Department of Mechanical Engineering, College of Engineering, Chubu University, 1200 Matsumoto-cho, Kasugai 487-8501, Japan

**Keywords:** micro/nano-scale flows, micro actuators, MEMS, direct simulation Monte Carlo (DSMC) method, computational fluid dynamics, rarefied gas, hydrodynamic trap

## Abstract

In this paper, we propose that thermally induced Knudsen forces in a rarefied gas can be exploited to achieve a tweezer-like mechanism that can be used to trap and grasp a micro-object without physical contact. Using the direct simulation Monte Carlo (DSMC) method, we showed that the proposed mechanism is achieved when a heated thin plate, mounted perpendicularly on a flat substrate, is placed close to a colder object; in this case, a beam. This mechanism is mainly due to the pressure differences induced by the thermal edge flows at the corners of the beam and the thermal edge flow at the tip of the thin plate. Specifically, the pressure on the top surface of the beam is smaller than that on its bottom surface when the thin plate is above the beam, while the pressure on the right side of the beam is smaller than that on its left side when the thin plate is located near the right side of the beam. These differences in pressure generate a force, which attracts the beam to the plate horizontally and vertically. Furthermore, this phenomenon is enhanced when the height of the beam is shorter, such that the horizontal and vertical net forces, which attract the beam to the plate, become stronger. The mechanism proposed here was also found to depend significantly on the height of the beam, the temperature difference between the thin plate and the beam, and the Knudsen number.

## 1. Introduction

In rarefied gas conditions, such as that in micro/nano-scale flows, an object placed close to a structure of a different temperature and receives a force [1]. This phenomenon is caused by temperature inhomogeneities in the gas at conditions where the Knudsen number, defined as the ratio of the mean free path for gas molecules to the characteristic length of the flow, is non-zero [2]. The class of forces induced in this phenomenon is called as Knudsen force. Recent advances in micro-electromechanical systems (MEMS) made it possible to fabricate micro/nano-devices that can exploit the Knudsen forces for specific applications [1,2,3,4,5,6,7,8,9,10]. Such technologies include microstructure actuation [5,6], gas sensing [7,8,9], atomic force microscopy [1,4], and high-precision force measurements [3], among others. In addition to experimental [2,10] and theoretical approaches [1,11,12,13], numerical approaches have also been used to study the Knudsen forces [7,10,14,15,16,17]. Computational studies are particularly significant to reproduce the thermally induced flow field in complex systems that is difficult to observe experimentally. The studies of [1,2,10,15,16,17,18] started from a relatively simple configuration; specifically, a microbeam was placed close to a flat plate. For this configuration, Nabeth et al. [15] and Zhu and Ye [16] provided their explanations for the mechanism of the Knudsen force. Specifically, the latter pointed out that the Knudsen force is caused mainly by the thermally induced flows in the bulk region of the gas, and that the configuration of the physical domain has a significant influence on the magnitude and direction of the Knudsen force. Therefore, the configuration can be manipulated with a desired application in mind, such as two beams of different temperatures placed close to each other to be used for gas sensor mechanism [5,6,7,9] and a heated object placed eccentrically inside a closed system to be used for rotational mechanism [12,13]. Furthermore, simple geometrical modifications in the actuator mechanism have shown better performance than conventional ones [19].

The above studies [1,2,3,4,5,6,7,8,9,10,11,12,13,14,15,16,17,18,19] proved that thermally induced Knudsen forces can displace an object without physical contact and any moving parts. This displacement is usually repulsive, such as a heated cantilever being deflected away from a colder flat substrate [1,2,8]. However, by reversing the temperature gradient, i.e., when a cold cantilever is placed close to a heated flat substrate, the direction of the generated force is reversed (attractive force) [4]. In this case, the thermally induced Knudsen force can displace the object closer to the substrate. Furthermore, if the substrate has a microstructure, the tangential Knudsen force will be induced on the object and can induce the self-propulsion of the object [11,14]. Combining these two ideas, it may be possible to use the thermally induced Knudsen forces to capture or manipulate an object without contact. Specifically, the usual configuration where a beam is placed close to a flat substrate can be modified to achieve a mechanism that can trap and manipulate a micro-object in a gas.

Contactless trapping and manipulating a micro-object suspended in a liquid has been extensively studied in microfluidics [20,21,22,23,24,25,26,27,28,29,30,31,32,33]. For example, optical trapping uses a laser beam as a tweezer-like mechanism, which can trap and directly manipulate micro-objects [20,21,22]. On the other hand, for objects that are sensitive to heat, such as biological cells, hydrodynamic trapping is another method where flow field is induced to trap and manipulate suspended micro-objects [23,24,25,26]. Alternatively, both mechanisms can also be employed together. For example, an optically trapped micro-rotor can induce highly localized flow fields that can be used to indirectly manipulate micro-objects [27]. Another example is the use of optical gradient and thermophoretic forces to trap targets to a focal spot [28,29]. However, these mechanisms have been limited to liquid conditions. Optical trapping of a micro-object is typically more complicated in gas than in water [30,31], while aerodynamic-based traps [32,33] have shown that artificially induced air flows can trap and manipulate objects similar to hydrodynamic traps. However, such systems use jets of air, and in those systems, high-precision manipulation of a micro-object is difficult to achieve. 

In this study, we take advantage of the thermally induced Knudsen force appearing uniquely in micro/nano-scale flows to propose a simple configuration of a tweezer-like mechanism, which can be used to trap and manipulate a micro-object in a gas. Specifically, we demonstrate that when a thin plate is perpendicularly mounted on a heated substrate and placed close to a colder beam (micro-object of interest), a force is generated on the beam and attracts the beam to the thin plate horizontally and vertically. Therefore, in this configuration, the thin plate acts like tweezers that can trap and manipulate the beam. Using the direct simulation Monte Carlo (DSMC) method, we reproduce the flow and study the Knudsen force induced on the beam. The effect of different parameters on the Knudsen force is also investigated in this paper.

## 2. Methodology

**Figure 1:**
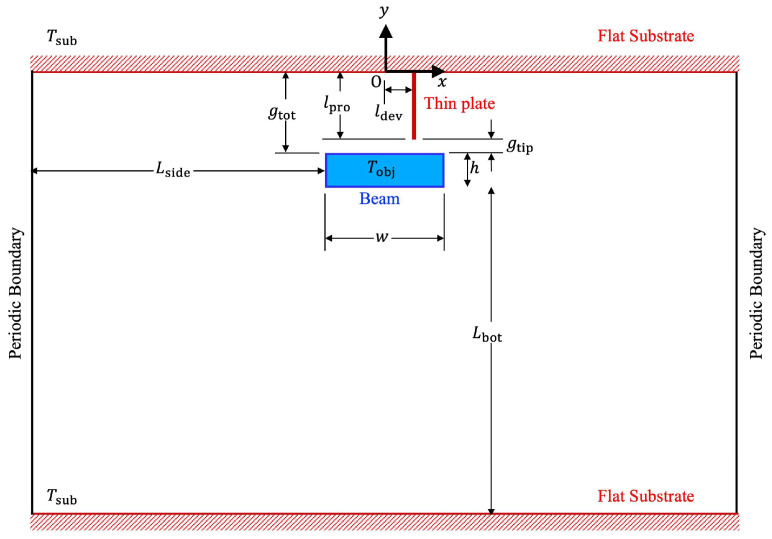
Schematic of the computational domain.

Let us consider the space between two horizontal infinitely wide flat substrates, which are filled with air. In this study, the function of tweezers is represented by a thin plate mounted perpendicularly on the upper substrate facing downward, and below the thin plate is the object to be grasped which is represented by a beam suspended in air, as shown in Figure 1. The two substrates and the thin plate are heated at a temperature $T_{\mathrm{sub}}=500$ K, while the object, i.e., the beam, is set at a room temperature $T_{\mathrm{obj}}=300$ K. Here, the width and the height of the beam are w and h, respectively, and its top surface is separated downward from the bottom surface of the flat upper substrate by a gap distance, $g_{\mathrm{tot}}$, as shown in Figure 1. Moreover, the thin plate is a protrusion of length $l_{\mathrm{pro}}$ from the substrate and is deviated rightward from the center of the beam by a distance of $l_{\mathrm{dev}}$. Therefore, the gap distance between the tip of the thin plate and the top surface of the beam, $g_{\mathrm{tip}}$, is given by $g_{\mathrm{tip}}=g_{\mathrm{tot}}-l_{\mathrm{pro}}$. Although the local flow field near the thin plate will be largely influenced by the size of the minimum gap $g_{\mathrm{tip}}$, the global flow field around the object will be determined by the size of the gap distance between the beam and the flat upper substrate, $g_{\mathrm{tot}}$. Therefore, in this study, the total gap distance, $g_{\mathrm{tot}}$, is considered as the characteristic length of the flow, which is used to calculate the Knudsen number, as mentioned later. 

Here, the x axis is set rightward and the y axis is set upward such that x=0 is colinear with the center of the beam, and such that y=0 is on the bottom surface of the upper substrate, i.e., the uppermost boundary. Furthermore, the computational domain is considered to be periodic in the −x and +x directions, and the bottommost boundary is considered to be a surface of the lower substrate. As shown in Figure 1, the distances between the left side of the computational domain and the left side of the beam and between the right side of the computational domain and the right side of the beam are both $L_{\mathrm{side}}$. The distance between the bottom surface of the beam and the bottommost boundary is $L_{\mathrm{bot}}$. 

In this study, air is used as the working gas. Although air is composed of molecules of different species, it is assumed here that air molecules are represented by imaginary hard-sphere molecules [34] with a mass $m=4.80967\times 10^{-26}$ kg. Note that, by using hard-sphere molecules of constant diameter instead of realistic air molecules, the discussion in this study can also be applied to other gases without being limited to air, provided that the Knudsen number is the same [14]. From the reference temperature at $T_{\mathrm{ref}}=\left(T_{\mathrm{sub}}+T_{\mathrm{obj}}\right)/2=400$ K, the equivalent viscosity is $\mu_{\mathrm{ref}}=2.301328\times 10^{-5}\;\mathrm{Pa}\cdot s$ [35,36]. From the relation $\mu_{\mathrm{ref}}=0.499mn_{\mathrm{ref}}{\bar{C}}_{\mathrm{ref}}\lambda_{\mathrm{ref}}$ and the mean free path for gas molecules given by $\lambda_{\mathrm{ref}}=1/\sqrt{2}n_{\mathrm{ref}}\pi d_{m}^{2}$, the diameter of the imaginary hard-sphere molecules is obtained and set at $d_{m}=0.356$ nm, where ${\bar{C}}_{\mathrm{ref}}$ is the average thermal speed of molecules and given by ${\bar{C}}_{\mathrm{ref}}=\sqrt{8k_{B}T_{\mathrm{ref}}/\pi m}$, $k_{B}$ is the Boltzmann constant given by $k_{B}=1.380649\times 10^{-23}\;J/K$, and $T_{\mathrm{ref}}$ is the number density of molecules at the reference temperature $T_{\mathrm{ref}}$ and the reference pressure $p_{\mathrm{ref}}$ and given by $n_{\mathrm{ref}}=p_{\mathrm{ref}}/k_{B}T_{\mathrm{ref}}$, and the reference pressure $p_{\mathrm{ref}}$ is set at the standard pressure $p_{\mathrm{ref}}=1$ atm. Using the above relation, for $d_{m}=0.356$ nm, the corresponding mean free path is $\lambda_{\mathrm{ref}}=96.79697\cdots$ nm, and the mean free time, $\tau_{\mathrm{ref}}=\lambda_{\mathrm{ref}}/{\bar{C}}_{\mathrm{ref}}$, is $\tau_{\mathrm{ref}}=1.790101\times 10^{-10}$ s. As mentioned above, in this study, the length $g_{\mathrm{tot}}$ is considered as the characteristic length, such that the Knudsen number, $\mathrm{Kn}=\lambda_{\mathrm{ref}}/g_{\mathrm{tot}}$. Furthermore, the reference lengths for the computational domain shown in Figure 1 are set relative to the reference mean free path $\lambda_{\mathrm{ref}}$, as tabulated in Table 1. Note that here, the Knudsen number is set at Kn=0.2, i.e., the length $g_{\mathrm{tot}}$ is set at $5\lambda_{\mathrm{ref}}$.

**Figure 2:**
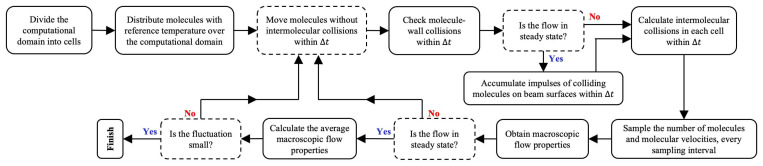
Flow chart of the DSMC method used in this study.

In this study, the focus is on non-continuum flow conditions, i.e., micro/nano-scale flows and, hence, the Navier–Stokes equation is not applicable. Here, the direct simulation Monte Carlo (DSMC) method [37,38] was used to simulate the flow. The DSMC method has already been used in several studies for the Knudsen forces on microbeams [7,16,17]. In the DSMC method, the motion of sample molecules, which are considered as a statistical representation of the real molecules, is simulated and tracked. Since the Knudsen force in a steady state is of interest in this study, macroscopic properties are then taken as the time average of the sampled molecular velocities. Free motions and intermolecular collisions are decoupled over a very small time step. In this study, the maximum collision number method [39,40] is used to treat intermolecular collisions. For details on the maximum collision number method used here, the reader is directed to ref. [14]. For simplicity, a surface accommodation coefficient of unity is assumed here, and the diffuse reflection model [34] is used for molecule–wall collisions. The DSMC method used in this study is described in the flow chart shown in Figure 2.

The physical domain in Figure 1 was divided into DSMC cells with uniform cell sizes Δx and Δy set smaller than the mean free path $\lambda_{\mathrm{ref}}$. For all the cases considered in this study, a cell size of $\Delta x=\Delta y=\lambda_{\mathrm{ref}}/4$ is used. Furthermore, the time step for the DSMC simulation was also set smaller than the mean free time $\tau_{\mathrm{ref}}$. For all the cases considered in this study, a time step of $\Delta t=0.08\tau_{\mathrm{ref}}$ is used. This chosen time step ensures that not only is it smaller than the mean free time, but also, no molecule can cross two cells within one time step. Moreover, sample molecules were initialized in each DSMC cell at a number of 30 for the standard cell size of Δx×Δy. The macroscopic flow properties are obtained by sampling the molecular velocities. For details on the calculation of the macroscopic properties, such as the number density n, the flow velocity v, and temperature T, in each DSMC cell, the reader is directed to ref. [14]. Of particular interest in this study is the force acting over the surfaces of the beam. The local surface force, $F_{i,l}$, per unit area is given by:

(1) $$\frac{F_{i,l}}{\Delta S_{l}}=\frac{W_{f}}{\Delta S_{l}\left(t_{\mathrm{end}}-t_{\mathrm{start}}\right)}\overset{N_{\mathrm{wall}-\mathrm{coll},l}}{\underset{q=1}{\sum }}m\left[{\left(\xi_{q}\right)}_{i}-{\left(\xi_{q}\right)}_{i}^{\prime }\right]$$

Here, $W_{f}$ is the number of real molecules represented by one sample molecule and $F_{i,l}$ is the ith component of the surface force exerted on the lth surface element with a width $\Delta S_{l}$ of the surface of the beam. In the present study, two-dimensional flow field is considered. Nevertheless, it means not that the thickness of the flow field is zero but that the flow field does not change in the z direction, which is normal to the xy plane. Thus, the thickness of the flow field in the z direction is regarded as unity. The same is true for the DSMC cell size. Using the volume of a DSMC cell with unit thickness, the factor $W_{f}$ was obtained by dividing the expected number of real molecules within the DSMC cell in the initial state by the number of initial sample molecules. Thus, the width $\Delta S_{l}$ represents the area of the lth surface element. While $N_{\mathrm{wall}-\mathrm{coll},l}$ is the number of sample molecules colliding onto the lth surface element during the sampling term from $t=t_{\mathrm{start}}$ to $t=t_{\mathrm{end}}$, $\xi_{q}$ and $\xi_{q}^{\prime }$ are the pre-collision and post-collision velocities of the qth sample molecule in terms of the order of wall–molecule collision occurring in the sampling term. The net force, $F_{i}$, is then obtained by integrating the local surface force per unit area $F_{i,l}/\Delta S_{l}$ in Equation (1) over the surface of the object.

## 3. Results and Discussion

### 3.1. Evaluation of the Proposed Device Compared to the Case of No Thin Plate

**Figure 3:**
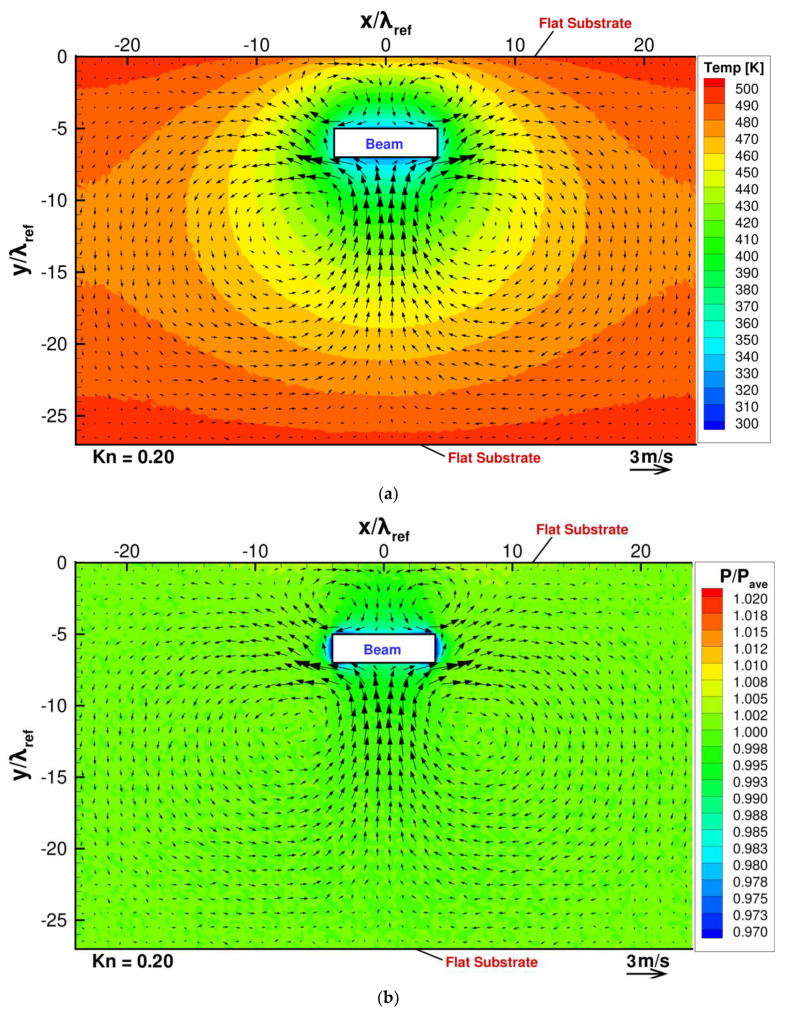
(**a**) Flow and temperature distribution and (**b**) pressure distribution, when the beam is placed close to a heated flat substrate, at Kn=0.2.

**Figure 4:**
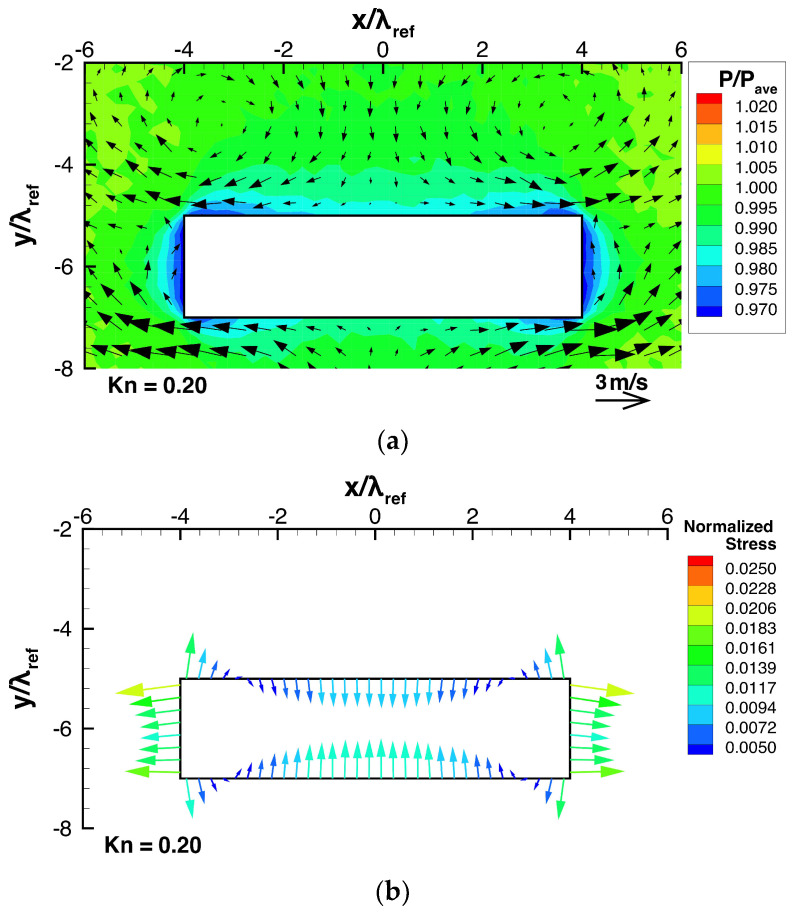
(**a**) Pressure and flow distributions around the beam and (**b**) stress distributions on the beam surfaces, when the beam is placed close to a heated flat substrate, at Kn=0.2.

Let us first consider the case when there is no thin plate mounted on the substrate at the uppermost boundary. Figure 3a,b show the temperature and pressure distributions with the flow distribution obtained in the DSMC simulation for such a case, and Figure 4a shows the flow and pressure distributions near the beam for the same case. Note that the pressure is normalized by $p_{\mathrm{ave}}$, which is the pressure spatially averaged in the whole region of the gas. In the present DSMC simulation, the gas pressure in the cell j is obtained by $p_{j}=n_{j}k_{B}T_{j}$, where $T_{j}$ is the temperature of gas in the cell j, $n_{j}$ is the number density of gas molecules in the cell j and given by $n_{j}=N_{j}/V_{j}$, $N_{j}$ is the number of real molecules in the cell j, and $V_{j}$ is the volume of the cell j. Here, the spatially averaged pressure $p_{\mathrm{ave}}$ is defined as $p_{\mathrm{ave}}=\left(\underset{j}{\sum }V_{j}p_{j}\right)/\left(\underset{j}{\sum }V_{j}\right)$, where $\underset{j}{\sum }\;$ represents the summation over all the DSMC cells. Since we define the spatially averaged number density and the spatially averaged temperature as $n_{\mathrm{ave}}=\left(\underset{j}{\sum }N_{j}\right)/\left(\underset{j}{\sum }V_{j}\right)$ and $T_{\mathrm{ave}}=\left(\underset{j}{\sum }N_{j}T_{j}\right)/\left(\underset{j}{\sum }N_{j}\right)$, respectively, the relation $p_{\mathrm{ave}}=n_{\mathrm{ave}}k_{B}T_{\mathrm{ave}}$ is satisfied. In the present simulations, the spatially averaged number density will not differ from the initial value, i.e., the setting value, because the number of real molecules will not vary during the simulations, and, hence, $n_{\mathrm{ave}}$ is constant and common for all the considered cases. Therefore, the spatially averaged pressure $p_{\mathrm{ave}}$ is proportional to the spatially averaged temperature $T_{\mathrm{ave}}$. On the other hand, the spatially averaged temperature $T_{\mathrm{ave}}$ is determined as a result of DSMC simulation and depends on the ratio of the area of hot surface to the area of the cold surface. Since this ratio depends on the cases, the resultant spatially averaged temperature varies depending on the cases. Due to the variation in $T_{\mathrm{ave}}$, the spatially averaged pressure $p_{\mathrm{ave}}$ also varies depending on the cases. Under the condition where the surrounding pressures are different among the cases, the pressure and the stress cannot be properly compared among the cases by using their absolute values. In order to avoid this problem, in the present study, we compare the pressure and the stress normalized by the spatially averaged pressure $p_{\mathrm{ave}}$ instead of their absolute values.

According to Sone, Y [41], in the case when an object of uniform temperature with sharp edges is immersed in a gas with a different temperature, the temperature field induced around the edges of the object drives a flow from a colder region to a hotter region near the edges. This flow is called thermal edge flow. The thermal edge flow should be considered as a pumping mechanism rather than a usual flow. For example, the driving mechanism of a pump using a thermal edge flow was proposed [42]. In the present study, the thermal edge flows are induced near the beam corners in the directions from the central region of each surface of the beam to the corners. Since the thermal edge flows play a role as pump, these flows move gas molecules away from the corners. As a result, low pressure is induced around the beam, as shown in Figure 3b. The low-pressure area on the bottom surface of the beam, which is induced by the thermal edge flow, sucks in gas from the wide bulk region of the gas below the beam due to the induced pressure difference; thus, the strong upward flow from the bulk to the beam is induced. This upward-pressure-driven flow supplies molecules to the low-pressure area on the bottom surface of the beam. In other words, this pressure-driven flow compensates the decrease in molecules in the central region of the bottom surface of the beam in such a manner that the supply of molecules is balanced with the removal of molecules due to the thermal edge flows in the steady state. Due to this compensation of the decrease in molecules by the upward-pressure-driven flow, the low pressure induced by the thermal edge flow is partly cancelled out, but remains in the central region of the bottom surface of the beam in the steady state, as shown in Figure 3b and Figure 4a. This is because if the low pressure is fully canceled out, the pressure-driven flow will disappear, and the thermal edge flow will pump out molecules from the considered region again and, hence, the flow cannot become steady.

The same is true for the low-pressure area on the top surface of the beam. The low-pressure region on the top surface of the beam sucks in gas from the narrow region of the gas above the beam. Thus, the downward-pressure-driven flow from the narrow region to the beam is induced. However, this downward flow to the top surface of the beam is much weaker than the upward flow to the bottom surface of the beam. The reason for the weaker downward flow is because the low-pressure area on the top surface can gather only a fewer number of molecules because of a smaller space between the beam and the upper substrate compared with the low-pressure area on the bottom surface. The decrease in the number of molecules near the bottom surface is more efficiently compensated by the strong upward molecular flow than that near the top surface. This causes relatively high pressure on the bottom surface compared with that on the top surface, as shown in Figure 3b and Figure 4a. This pressure difference can be seen in the stress distribution exerted on the beam surface shown in Figure 4b.

After the upward-pressure-driven flow impinges on the bottom surface of the beam, it is directed sideways. As a result, it forms two vortices of opposite rotation, such that it satisfies the equation of continuity. However, the formation of two vortices seen here can be considered to be caused by the limited region of the computational domain in the present study. If the domain is large enough, i.e., if the lower substrate and the periodic boundaries on the right and left sides are far enough from the beam, it can result in just a jet of gas impinging on a plate, which is converted into two opposing flows along the plate. In the same manner, two opposing vortices are induced also near the top surface of the beam, although they are much weaker than those appearing near the bottom surface. This is because the downward-pressure-driven flow to the top surface of the beam is much weaker than the upward-pressure-driven flow to the bottom surface of the beam, as mentioned above. 

**Figure 5:**
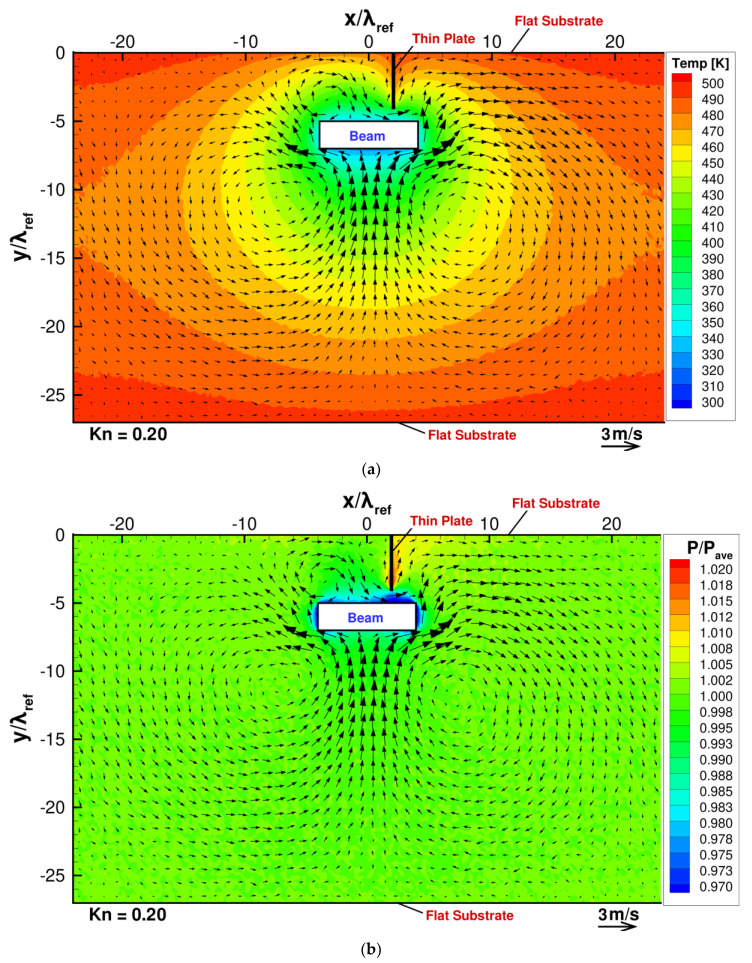
(**a**) Flow and temperature distribution and (**b**) pressure distribution, when the beam is placed close to a heated thin plate mounted on a substrate, at Kn=0.2.

**Figure 6:**
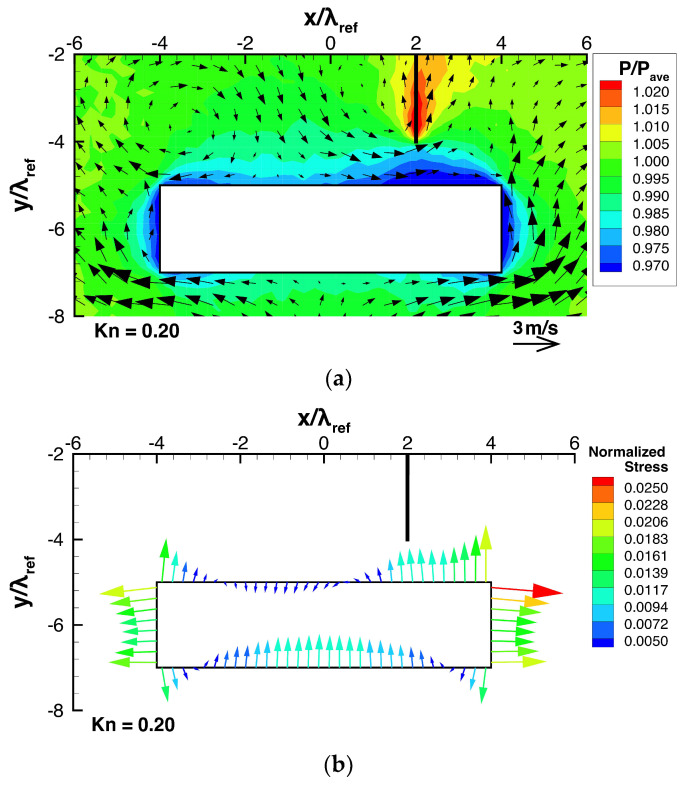
(**a**) Pressure and flow distributions around the beam and (**b**) stress distributions on the beam surfaces, when the beam is placed close to a heated thin plate mounted on a substrate, at Kn=0.2.

Let us now consider the case when a thin plate is mounted on a flat substrate. Figure 5a,b show the temperature and pressure distributions with the flow distribution obtained from DSMC for such a case, and Figure 6a shows the flow and pressure distributions near the beam for the same case. In this case, except the region near the right side of the top surface of the beam, the obtained flow field is similar to the case of no thin plate, while the right vortex near the top surface of the beam naturally disappears because it is obstructed by the thin plate. Since the thin plate is hotter than the surrounding gas, a thermal edge flow is induced along the plate from the gap region between its tip and the top surface of the beam towards its root. Thus, similar to the thermal edge flow appearing around the corners of the beam, the thermal edge flow appearing around the edge of the thin plate acts like a pump that sucks in gas from the region near the top surface of the beam. This results in a reduction in pressure on the top surface of the beam located near the thin plate, as seen in Figure 6a. As a result, this increases the pressure difference between the bottom and top surfaces of the beam. Furthermore, since this hot thin plate is located near the right top corner of the cold beam, the temperature gradient near the right top corner is increased, as seen in Figure 5a. Due to this, the upward thermal edge flow along the right side of the beam is enhanced, as seen in Figure 5a and Figure 6a. This enhanced thermal edge flow along the right side of the beam carries molecules out of the region on the right side and, hence, induces the reduction in pressure on the right side of the beam, as seen in Figure 6a, whereas the normalized pressure on the left side is the same as that for the case of no plate shown in Figure 4a. In the case of no thin plate, the pressures on the right and left sides are the same because of symmetry in the field and, hence, no horizontal force appears. On the other hand, in the case when the thin plate is mounted and deviated rightward from the center of the beam, a rightward force is induced by the above-mentioned pressure difference appearing on the right and left sides of the beam.

Usually, in a gas, absolute values for the normal components of stress are much larger than those of its tangential components. This is because the normal component of stress includes the contribution of ambient pressure. If we draw a figure of stress exerted on the beam surface, such as Figure 4b, using the absolute stresses exerted on it, the tangential components of stresses are not visible since their absolute values are much smaller than those of normal components. Nevertheless, in general, if we integrate a surface force due to ambient pressure or some other constant pressure over a surface of an object, the net force exerted on the object due to such pressure is zero. Therefore, the contribution of ambient pressure or some other constant pressure can be excluded when we discuss the net force exerted on the object. Considering this fact, it is convenient for us to subtract some constant pressure comparable to ambient pressure from the normal components of the local stress when we discuss the net force exerted on the beam. Due to this subtraction, the magnitudes of the normal components of stress can be expressed in the same order as those of the tangential components of stress. Here, firstly, we calculate the average pressure exerted on the beam surface, ${\bar{p}}_{\mathrm{surface}}$, which is obtained by averaging a pressure obtained by Equation (1) over the beam surface, and then divide this superficially averaged pressure ${\bar{p}}_{\mathrm{surface}}$ by the spatially averaged pressure $p_{\mathrm{ave}}$. Then, we subtract the obtained value from the normal component of the stress obtained by Equation (1), which is also normalized by $p_{\mathrm{ave}}$. Thus, we obtain local stresses relative to the superficially averaged pressure ${\bar{p}}_{\mathrm{surface}}$ exerted on the beam surface as follows:

(2) $$\mathrm{Normalized}\;\mathrm{modified}\;\mathrm{normal}\;\mathrm{stress}=\frac{\mathrm{normal}\;\mathrm{stress}\;}{p_{\mathrm{ave}}}-\frac{{\bar{\;p}}_{\mathrm{surface}}\;}{p_{\mathrm{ave}}}$$

(3) $$\mathrm{Normalized}\;\mathrm{modified}\;\mathrm{tangential}\;\mathrm{stress}=\frac{\mathrm{tangential}\;\mathrm{stress}}{p_{\mathrm{ave}}}$$

Here, in order to differentiate the thus-obtained stress from absolute pressure or absolute stress, we call it “modified stress”, of which the concept is similar to “gauge pressure”. Furthermore, note that in the present study, “normal stress” is reckoned as positive when it corresponds to a state of compression. 

Figure 4b shows the distribution of the modified stress exerted on the beam surface in the case of no thin plate. In this case, the ratio ${\bar{p}}_{\mathrm{surface}}/p_{\mathrm{ave}}$ was 0.9813. Here, the normal component of normalized modified stress has a value in the range $-2\times 10^{-2}$ to $2\times 10^{-2}$ as shown in Figure 4b. In the case of negative value, the modified stress is drawn as if it pulls the beam outward like a tension in Figure 4b. However, the magnitudes of normalized modified stresses are much smaller than the subtracted normalized pressure of 0.9813. Therefore, the tensile stresses in Figure 4b should not be considered real tension. Actually, they represent a positive but low pressure, less than $0.9813\;p_{\mathrm{ave}}$. On the other hand, the compressive stresses in Figure 4b represent a positive pressure higher than $0.9813\;p_{\mathrm{ave}}$. In Figure 6b, we show the stress distribution such as Figure 4b for different case, where the same subtracted value as that for the case of no thin plate in Figure 4b, i.e., 0.9813, was used. The same process is done for the stress distributions like Figure 4b presented in Section 3.2 and Section 3.3. In Figure 4b, we chose the superficially averaged pressure, ${\bar{p}}_{\mathrm{surface}}$, as a subtracted pressure to visualize the effective force due to stress on the beam. However, for the purpose of examining the net force on the beam, we can choose subtracted pressure flexibly, in a way that is convenient for us. Therefore, here, we choose the same subtracted normalized pressure of 0.9813 for all the cases. Thanks to this choice, we can directly compare the normalized modified stress over all the cases.

In the case when there is no thin plate mounted on the substrate, as shown in Figure 4b, the stresses are symmetric in the x-direction; hence, zero net force in the x-direction can be obtained. However, the magnitude of the pressure exerted on the bottom surface of the beam is slightly larger than that exerted on the top surface of the beam; hence, a non-zero net force is directed upward in the y-direction. This pressure difference can be seen also in the pressure distribution in Figure 3b and Figure 4a, as mentioned above. 

On the other hand, in the case when a thin plate is mounted on the substrate, as shown in Figure 6b, the stresses are non-uniform and asymmetric horizontally and vertically. Specifically, as mentioned in the discussion for Figure 6a, the stresses on the left and right sides of the beam are unequal, which result in a non-zero net force directed rightward. Furthermore, the presence of the thin plate causes an increase in the difference between the normal stresses exerted on the bottom and top surfaces of the beam, as mentioned above; hence, a stronger net force directed upward is obtained. Particularly, the upward force is stronger in the vicinity of the thin plate compared to the case of no thin plate shown in Figure 4b. Here, note that the upward pulling force drawn in the vicinity of the thin plate represents not a real tension but a positive pressure, significantly smaller than the other parts of the beam surface, as mentioned above. These non-zero net forces are caused by the pressure differences on the surfaces of the beam, whose mechanism is described in the fifth paragraph of this section. The results show here that by mounting a thin plate on a flat substrate, a force that attracts the beam to the plate horizontally and vertically is induced. 

### 3.2. Effect of the Position of the Thin Plate

**Figure 7:**
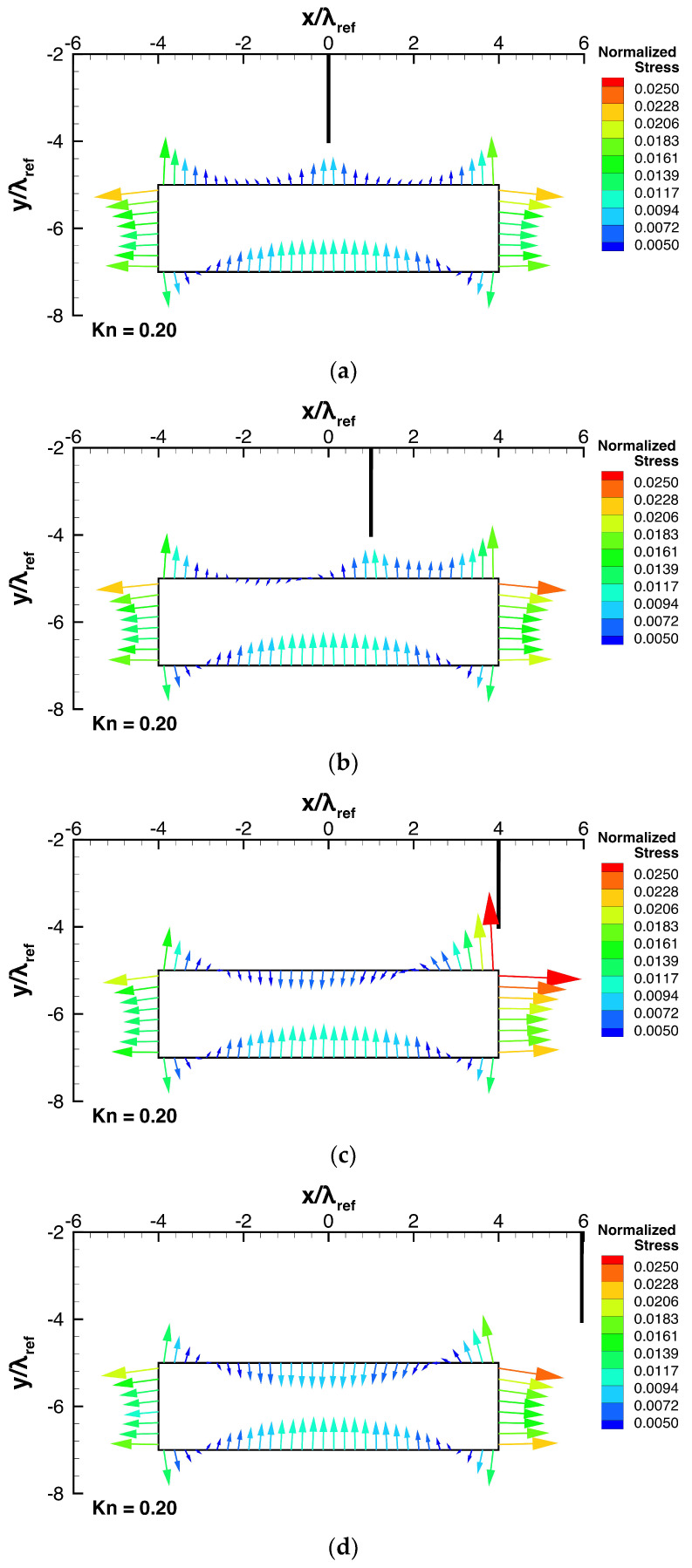
Stress distributions for different deviation distances, $l_{\mathrm{dev}}$, at Kn=0.2. (**a**) $l_{\mathrm{dev}}=0$ . (**b**) $l_{\mathrm{dev}}=0.125w$. (**c**) $l_{\mathrm{dev}}=0.5w$. (**d**) $l_{\mathrm{dev}}=0.75w$.

**Figure 8:**
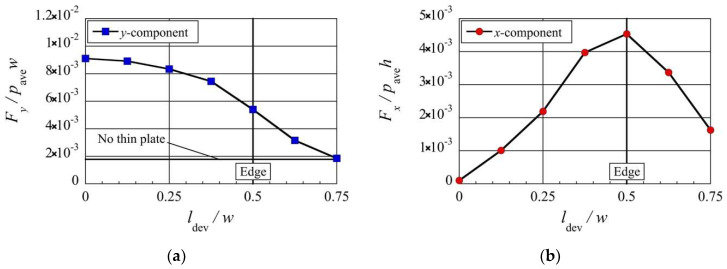
Net (**a**) upward and (**b**) rightward forces exerted on the beam for different deviation distances, $l_{\mathrm{dev}}$, at Kn=0.2.

Next, let us consider how the position of the thin plate relative to the beam affects the stress and the resultant net force exerted on the beam, by adjusting the deviation distance, $l_{\mathrm{dev}}$, while keeping all other parameters constant. Figure 7a–d show the stress distributions on the beam surface for such cases. Figure 8 shows the dependencies of the resultant net rightward and upward forces, $F_{x}$ and $F_{y}$, exerted on the beam on the deviation distance, $l_{\mathrm{dev}}$. Here, the rightward and upward forces, $F_{x}$ and $F_{y}$, are normalized by the forces exerted on the left side and the bottom surface due to the spatially averaged pressure $p_{\mathrm{ave}}$, i.e., $p_{\mathrm{ave}}\times h$ and $p_{\mathrm{ave}}\times w$, respectively.

Consider the case of no thin plate shown in Figure 4b as a reference case. By mounting a thin plate on the substrate just above the center of the beam, as shown in Figure 7a, the high pressure on the central region of the top surface of the beam shown in Figure 4b is weakened, while the stresses exerted on the other surfaces are almost the same as those of the case of no thin plate. This results in the strong upward net force, as shown in Figure 8a. This net upward force is 0.009-times as strong as the force due to the spatially average pressure $p_{\mathrm{ave}}$. In the case when the system is in the atmospheric air condition, a lift force of about 900 Pa can be obtained. Assuming that the beam is of silicon material (2329 $\mathrm{kg}/m^{3}$ [36]), this lift force is equivalent to the force that levitates the beam with a height of 40 mm.

As the position of the thin plate is moved rightward away from the center of the beam, the region on the top surface of the beam where the pressure is reduced by the upward thermal edge flow towards the thin plate moves rightwards together with the position of the thin plate, as shown in Figure 6b and Figure 7a–d. Since the region of pressure reduction is gradually deviated from the center of the beam as the thin plate moves rightward from the center of the beam, the net upward force decreases with increasing $l_{\mathrm{dev}}$, as shown in Figure 8a. Note that even in the case of no thin plate, the net upward force is non-zero. It is indicated by a horizontal solid line in Figure 8a. In Figure 8a, the position of the edge of the beam, x=0.5w, i.e., $x=4\lambda_{\mathrm{ref}}$, is also indicated by the vertical solid line. By mounting a thin plate on the flat substrate, the net upward force is enhanced, as shown in Figure 8a. However, this is only true up to the point when the position of the thin plate is at $l_{\mathrm{dev}}=0.75w$ from the center of the beam, where the net upward force approaches that of the case of no thin plate. Note that in the case of $l_{\mathrm{dev}}=0.75w$, the thin plate is located outside the region above the beam, i.e., −0.5w≤x≤0.5w.

Simultaneously, as the thin plate is moved towards the edge of the beam from its center, the pressure on the right side of the beam is gradually reduced, as shown in Figure 6b and Figure 7a–c. As a result, the net rightward force appears and increases with increasing $l_{\mathrm{dev}}$, up to the edge of the beam, i.e., $0<l_{\mathrm{dev}}\leq 0.5w$, as shown in Figure 8b. Similarly to Figure 8a, the position of the edge of the beam, x=0.5w, is indicated in Figure 8b. The net rightward force obtained when the thin plate is located at the right edge of the beam is 0.0045-times as strong as the force due to the spatially average pressure $p_{\mathrm{ave}}$. In the case when the system is in the atmospheric air condition, a rightward force of about 450 Pa can be obtained. Assuming that the beam is of silicon material, this force is equivalent to the force that accelerates the beam with a width of 20 mm rightward with the earth’s gravitational acceleration. However, when the position of the thin plate is moved further away from the edge of the beam, the reduction in pressure on the right side of the beam is decreased, as shown in Figure 7d. As a result, the net rightward force decreases as the thin plate is moved further away from the edge of the beam, i.e., $l_{\mathrm{dev}}>0.5w$, as shown in Figure 8b. 

Nevertheless, the results here show that in a range of $0<l_{\mathrm{dev}}\leq 0.75w$, the net force is always directed upward and rightward, i.e., it always attracts the beam to the plate horizontally and vertically. Therefore, by using these thermally induced forces, the mechanism where the thin plate acts like tweezers that can trap the beam is obtained.

### 3.3. Effect of the Beam Height

**Figure 9:**
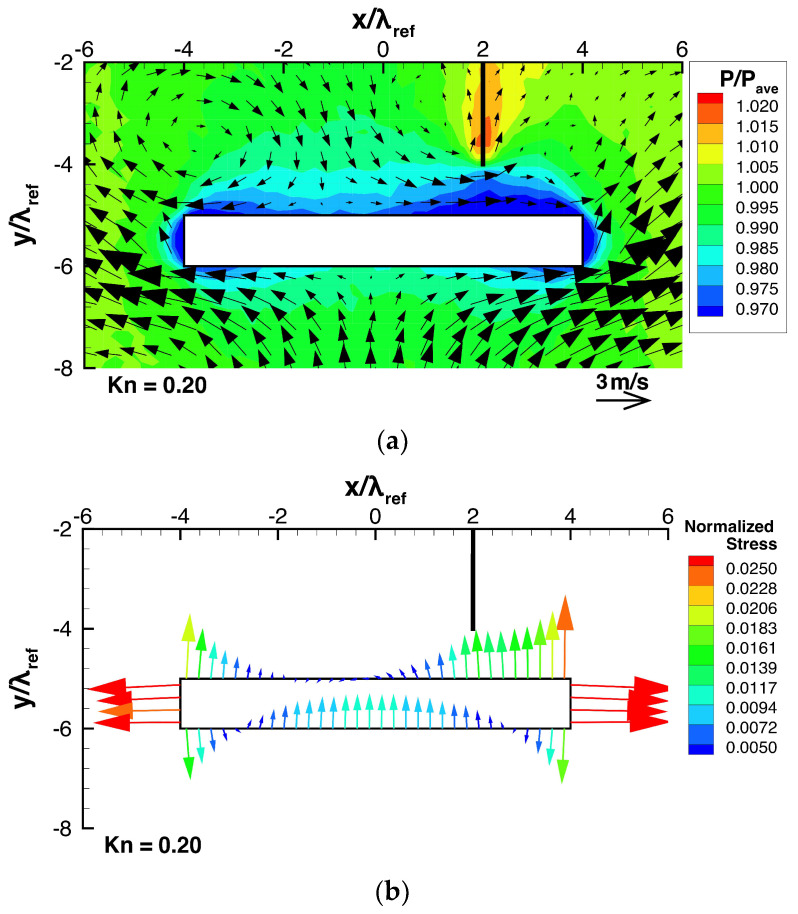
(**a**) Pressure and flow distributions around the beam and (**b**) stress distributions on the beam surfaces for a beam height of h=0.125w, at Kn=0.2.

**Figure 10:**
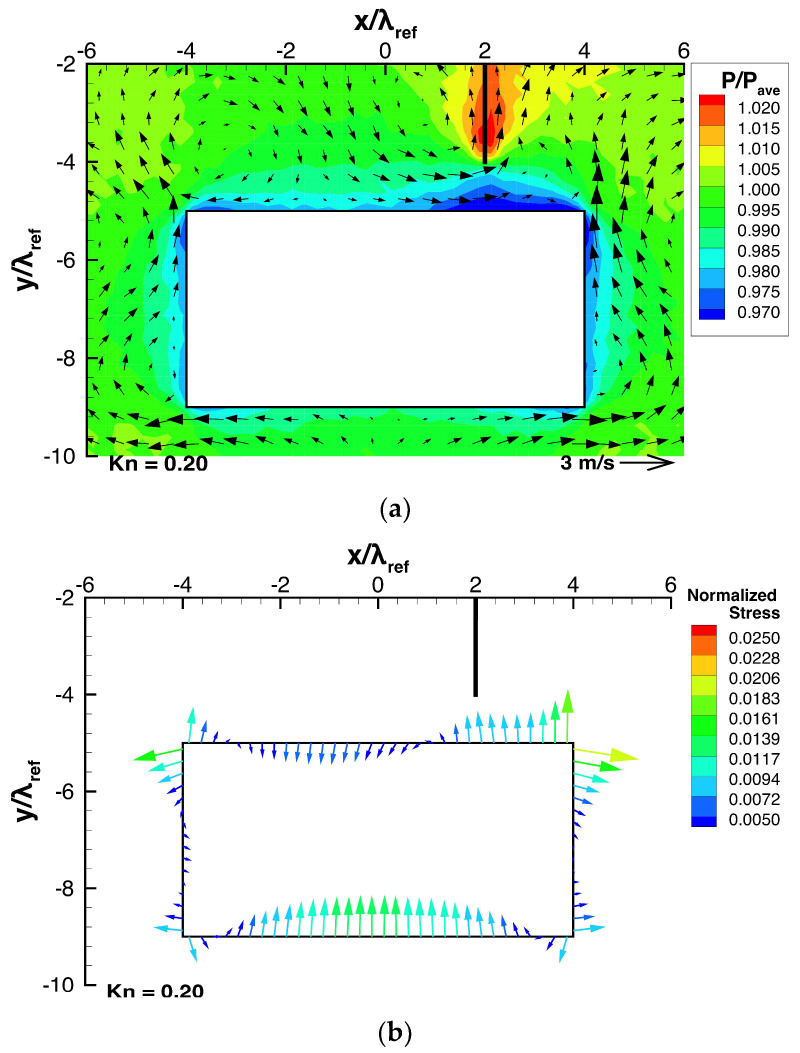
(**a**) Pressure and flow distributions around the beam and (**b**) stress distributions on the beam surfaces for a beam height of h=0.375w, at Kn=0.2.

**Figure 11:**
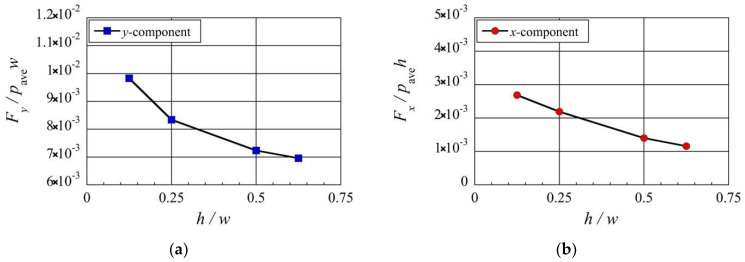
Net (**a**) upward and (**b**) rightward forces exerted on the four different beam heights, h, at Kn=0.2.

Next, let us investigate the effect of the height of the beam, h, on the stress and the resultant net force exerted on the beam, by adjusting the beam height, h, while keeping all other parameters constant. For such cases, Figure 9a and Figure 10a show the flow and pressure distributions near the beam, and Figure 9b and Figure 10b show the stress distributions on the beam surface. Figure 11 shows the dependencies of the resultant net rightward and upward forces, $F_{x}$ and $F_{y}$, exerted on the beam on the height. Here, similar to what was shown in Section 3.1 and Section 3.2, the rightward and upward forces, $F_{x}$ and $F_{y}$, are normalized by the forces exerted on the left side and the bottom surface due to the spatially averaged pressure $p_{\mathrm{ave}}$, i.e., $p_{\mathrm{ave}}\times h$ and $p_{\mathrm{ave}}\times w$, respectively. 

Consider the case shown in Figure 6, where h=0.25w and $l_{\mathrm{dev}}=0.25w$, as the reference case. Firstly, let us compare the case when the beam height is decreased to h=0.125 w as shown in Figure 9, with the reference case. By decreasing the beam height, the thermal edge flows around the corners of the beam become stronger and the pressure on the top surface of the beam is decreased, as shown in Figure 9a, compared to Figure 6a. However, the high pressure on the central region of the bottom surface of the beam is almost the same as that of the reference case. As a result, the pressure difference between the bottom and top surfaces of the beam is increased, as seen in Figure 6a,b and Figure 9a,b. Therefore, a stronger upward net force is generated, as shown in Figure 11a. Simultaneously, as the beam height is decreased, the pressures on both the right side and the left side of the beam are reduced, as shown in Figure 6a and Figure 9a. Although the pressure is decreased on both sides, the pressure reduction on the right side is slightly greater than that on the left side, as shown in Figure 6a,b and Figure 9a,b. Therefore, a stronger rightward net force is generated, as shown in Figure 11b.

Secondly, let us compare the case when the beam height is changed to h=0.375w, with the reference case. By increasing the beam height, as shown in Figure 10a, the thermal edge flows around the corners of the beam become weaker and the reduction in the pressure on the top surface of the beam is decreased compared to Figure 6a. However, the high pressure on the central region of the bottom surface of the beam remains almost the same as that of the reference case. As a result, the pressure difference between the bottom and top surfaces of the beam is decreased, as seen in Figure 6a,b and Figure 10a,b. Therefore, upward net force is weakened, as shown in Figure 11a. Simultaneously, as the beam height is increased, the reductions in the pressures on both the right side and the left side of the beam are decreased, as shown in Figure 6a and Figure 10a. The decrease in the reductions in the pressures on both sides indicates that the pressures on both sides become closer to the average pressure and, hence, the pressure difference between both sides becomes smaller. Therefore, a rightward net force is still generated, but weaker, as shown in Figure 11b. The results presented here and in the previous paragraph show that for a shorter beam height, the net upward and rightward forces are stronger.

### 3.4. Effect of Lower Temperature Differences

**Figure 12:**
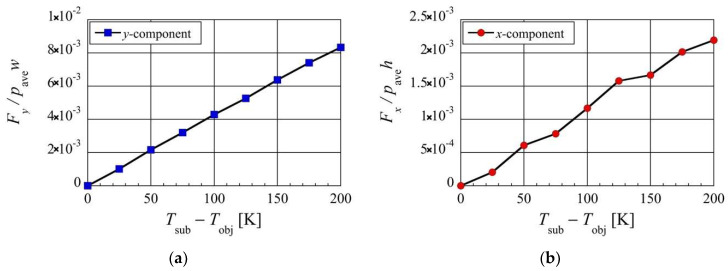
Net (**a**) upward and (**b**) rightward forces exerted on the beam at different temperature differences, in the case of Kn=0.2 and $\left(T_{\mathrm{sub}}+T_{\mathrm{obj}}\right)/2=400\;K$.

So far, only a temperature difference of 200 K has been considered. Now, let us consider the effect of using temperature differences lower than 200 K between the heated substrate (thin plate) and the colder object (beam), i.e., $T_{\mathrm{sub}}-T_{\mathrm{obj}}$, on the net forces. Figure 12 shows the distribution of the resultant net rightward and upward forces, $F_{x}$ and $F_{y}$, exerted on the beam, for various temperature differences less than and equal to 200 K at Kn=0.2. Here, the temperature difference $T_{\mathrm{sub}}-T_{\mathrm{obj}}$ is changed while the middle value $\left(T_{\mathrm{sub}}+T_{\mathrm{obj}}\right)/2$ is kept at 400 K. Similar to what was described in Section 3.2 and Section 3.3, the rightward and upward forces, $F_{x}$ and $F_{y}$, are normalized by the forces exerted on the left side and the bottom surface due to the spatially averaged pressure $p_{\mathrm{ave}}$, i.e., $p_{\mathrm{ave}}\times h$ and $p_{\mathrm{ave}}\times w$, respectively. It can be seen from Figure 12 that as the temperature difference is decreased from 200 K, both the net upward and rightward forces decrease. Specifically, both the net upward and rightward forces are proportional to the temperature difference, and both vanish in the case of no temperature difference, i.e., $T_{\mathrm{sub}}=T_{\mathrm{obj}}$. As discussed in previous sections, the reduction in pressure around the surface of the beam is due to the thermal edge pump effect. A low-temperature difference means that the strength of the thermal edge flows is weak. This results in smaller reductions in pressure around the beam and, hence, weaker net upward and rightward forces. In the case of the minimum temperature difference considered here at 25 K, under the atmospheric air condition, a lift force of about 100 Pa and a rightward force of about 20 Pa can be obtained. Assuming that the beam of silicon material of the density of $2329\;\mathrm{kg}/m^{3}$, this lift force is equivalent to the force that levitates the beam with a height of 4 mm, and the rightward force is equivalent to the force that accelerates the beam with a width of 0.8 mm rightward with the Earth’s gravitational acceleration. Thus, the obtained force for low-temperature differences is still large enough to move a small object less than 1 mm. Therefore, even at the low temperature difference of 25 K, the mechanism proposed in this paper can still be achieved.

### 3.5. Effect of Knudsen Number

**Figure 13:**
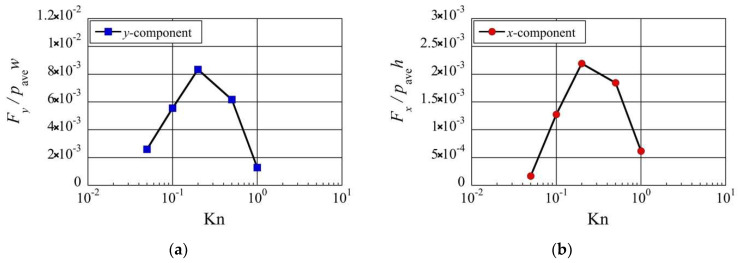
Net (**a**) upward and (**b**) rightward forces exerted on the beam at different Knudsen numbers.

Up to this point, only a Knudsen number of Kn=0.2 was considered. Now, let us consider the effect of different Knudsen number cases on the net forces. Figure 13 shows the resultant net rightward and upward forces, $F_{x}$ and $F_{y}$, exerted on the beam, for various Knudsen numbers. Here, the Knudsen number is adjusted by adjusting all the lengths of the setup considered here while keeping the gas mean free path $\lambda_{\mathrm{ref}}$ constant, i.e., keeping the reference gas pressure $p_{\mathrm{ref}}$ constant. For example, the total gap distance $g_{\mathrm{tot}}$ is adjusted as $g_{\mathrm{tot}}=\lambda_{\mathrm{ref}}/\mathrm{Kn}$. Similar to what was described in Section 3.2, Section 3.3 and Section 3.4, the rightward and upward forces, $F_{x}$ and $F_{y}$, are normalized by the forces exerted on the left side and the bottom surface due to the spatially averaged pressure $p_{\mathrm{ave}}$, i.e., $p_{\mathrm{ave}}\times h$ and $p_{\mathrm{ave}}\times w$, respectively. It can be seen from Figure 13 that the net upward force and the net rightward force are both maximum around Kn≈0.2, which is used as the reference Kn throughout this study, and both become small at low Knudsen numbers Kn<0.05 and high Knudsen numbers Kn>1. Note that the Knudsen number here is evaluated based on the total gap distance, $g_{\mathrm{tot}}$, such that $\mathrm{Kn}=\lambda_{\mathrm{ref}}/g_{\mathrm{tot}}$. 

As discussed in previous sections, the reduction in pressure around the surface of the beam is due to the thermal edge pump effect. Specifically, the low-pressure region on the top surface of the beam is increased by the thermal edge flow induced on the tip of the thin plate. Therefore, considering the local Knudsen number between the tip of the thin plate and the top surface of the beam, i.e., ${\mathrm{Kn}}_{\mathrm{tip}}=\lambda_{\mathrm{ref}}/g_{\mathrm{tip}}$, is significant when discussing the effect of the Knudsen number on the net forces. Note that ${\mathrm{Kn}}_{\mathrm{tip}}=5\mathrm{Kn}$ since $g_{\mathrm{tip}}=g_{\mathrm{tot}}/5$. Using ${\mathrm{Kn}}_{\mathrm{tip}}$, we can restate the above-mentioned knowledge as follows: the net upward force and the net rightward force are both maximum around ${\mathrm{Kn}}_{\mathrm{tip}}\approx 1$ and both become small at low Knudsen numbers ${\mathrm{Kn}}_{\mathrm{tip}}<0.25$ and high Knudsen numbers ${\mathrm{Kn}}_{\mathrm{tip}}>5$. This means that the thermally induced forces, which attract the beam to the thin plate horizontally and vertically, are optimum when the distance between the beam and the thin plate is in the order of one mean free path, as what is used throughout this study.

### 3.6. Summary of the Mechanism

**Figure 14:**
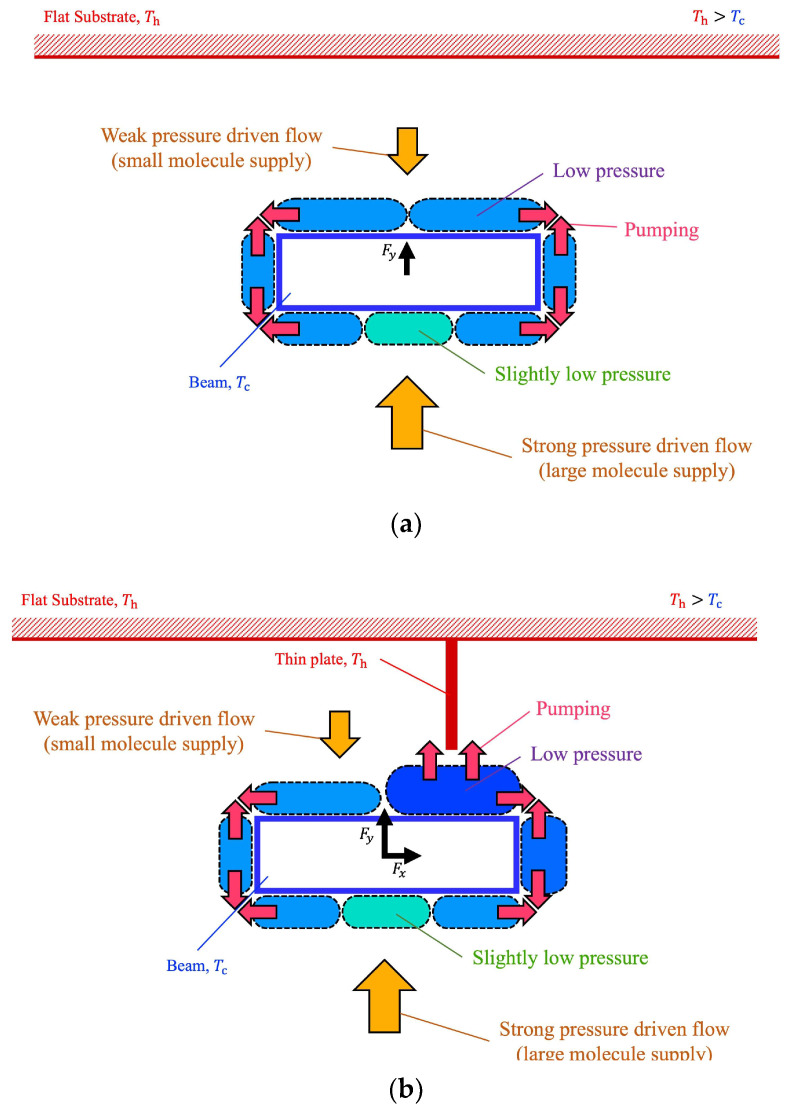
Schematic of the mechanism of the thermally induced forces on the beam (**a**) when the beam is placed close to a flat substrate and (**b**) when the beam is placed close to a heated thin plate mounted on a substrate.

Finally, we can summarize the mechanism of the generation of Knudsen forces for the configuration proposed here, and how it can be exploited to trap and manipulate a micro-object (beam). In the vicinity of the corners of the isothermal beam, the isothermal surfaces in the gas are largely curved to surround the sharp corners. Such a temperature distribution gives rise to thermal edge flows that result in the thermal edge pump phenomenon. This pumping effect moves molecules away from the region around the beam surfaces and then generates low-pressure regions around the beam surfaces. As a result, pressure-driven flows appear to compensate the decrease in molecules in the low-pressure regions, as illustrated in Figure 14a. The low-pressure region on the top surface of the beam is more weakly compensated than the low-pressure region on the bottom surface of the beam, since only a smaller number of molecules can be gathered in the narrow space between the top surface of the beam and the upper substrate than the wider space between the bottom surface of the beam and the lower substrate. This results in lower pressure on the top surface of the beam than that on the bottom surface of the beam. This pressure difference results in a net upward force, as illustrated in Figure 14a. 

When a thin plate is mounted perpendicularly on the flat substrate, the thermal edge pump phenomenon is enhanced by the additional thermal edge flow at the tip of the thin plate. This additional pumping effect enhances the pressure reduction on the top surface of the beam, as illustrated in Figure 14b. Furthermore, if the thin plate deviates from the center of the beam, i.e., it is closer to the right edge of the beam, the pressure reduction on the right side of the beam is enhanced, as illustrated in Figure 14b. Meanwhile, the pressures at the bottom surface and left side of the beam are unaffected, whether there is a thin plate or not. In this case, a pressure difference between the left and right sides of the beam is generated, and the pressure difference between the top and bottom surfaces of the beam is increased, as illustrated in Figure 14b. This results in a net rightward force and a stronger net upward force. Therefore, using the induced Knudsen forces here, the thin plate acts as a tweezer-like mechanism that can trap and manipulate the beam (micro-object) without contact. Note that as the Knudsen force depends on factors, such as the temperature difference, the Knudsen number, and the shape of the configuration, e.g., the geometry of micro-object; the strength and behavior of the phenomenon discussed here also depends on such factors.

## 4. Conclusions

In this study, we propose that the phenomenon of thermally induced Knudsen forces can be exploited to manipulate a micro-object. Specifically, a concept of a tweezer-like mechanism that can trap and grasp a micro-object without contact was presented. Using the DSMC method, it was demonstrated that the proposed mechanism is achieved when a heated thin plate mounted perpendicularly on a flat substrate is placed close to a micro-object, which, in this case, is a beam. The driving mechanism is mainly due to the pressure differences induced by the thermal edge flows at the corners of the beam and the thermal edge flow at the tip of the thin plate. These flows act like pumps that move gas molecules from the colder regions to the hotter regions around the edges. When the thin plate is above the beam, the pressure on the top surface of the beam is lower than that on its bottom surface. Furthermore, when the thin plate is located away from the center of the beam and is near the right side of the beam, the pressure on the right side of the beam is lower than that on its left side. Due to these pressure differences between the top and bottom surfaces and between the left and right sides of the beam, a force that attracts the beam to the plate horizontally and vertically is induced. On the other hand, in the case when the height of the beam is shorter, this phenomenon is enhanced, such that a stronger net upward and rightward force is induced. Furthermore, both the net upward and rightward forces were found to be proportional to the temperature difference, and both vanish in the case of no temperature difference. However, even at the low-temperature difference of 25 K, the obtained force is still large enough to move a small object less than 1 mm and, hence, the mechanism proposed in this paper can still be achieved. Moreover, we also found that these thermally induced Knudsen forces are optimum when the distance between the beam and the thin plate is in the order of one mean free path. In this study, we focused on a beam as the micro-object of interest. Whether the mechanism proposed here also applies to other shapes, such as a sphere, remains in question and will be a subject of future research.

## Figures and Tables

**Table 1:** Reference lengths for the physical domain.

Parameter	Symbol	Value
Beam width	w	$8\lambda_{\mathrm{ref}}$
Beam height	h	$2\lambda_{\mathrm{ref}}$
Length of protrusion	$l_{\mathrm{pro}}$	$4\lambda_{\mathrm{ref}}$
Tip to beam distance	$g_{\mathrm{tip}}$	$\lambda_{\mathrm{ref}}$
Total gap	$g_{\mathrm{tot}}$	$5\lambda_{\mathrm{ref}}$
Deviation of plate from center	$l_{\mathrm{dev}}$	$2\lambda_{\mathrm{ref}}$
Distance of side boundaries	$L_{\mathrm{side}}$	$20\lambda_{\mathrm{ref}}$
Distance of bottommost boundary	$L_{\mathrm{bot}}$	$20\lambda_{\mathrm{ref}}$

## Data Availability

Data sources can be provided upon request.

## References

[1] Passian A., Wig A., Meriaudeau F., Ferrel T.L., Thundat T. (2002). Knudsen forces on microcantilevers. J. Appl. Phys..

[2] Lereu A.L., Passian A., Warmack R.J., Ferrell T.L., Thundat T. (2004). Effect of thermal variations on the Knudsen forces in the transitional regime. Appl. Phys. Lett..

[3] Harris B.W., Chen F., Mohideen U. (2000). Precision measurement of the Casimir force using gold surfaces. Phys. Rev. A.

[4] Gotsmann B., Dürig U. (2005). Experimental observation of attractive and repulsive thermal forces on microcantilevers. Appl. Phys. Lett..

[5] Strongrich A., Alexeenko A. (2015). Microstructure actuation and gas sensing by the Knudsen thermal force. Appl. Phys. Lett..

[6] Alexeenko A., Strongrich A., Cofer A.G., Pikus A., Sebastiao I.B., Tholeti S.S., Shivkumar G. (2016). Microdevices enabled by rarefied flow phenomena. AIP Conf. Proc..

[7] Hassanvand A., Gerdroodbary M.B., Moradi R., Amini Y. (2018). Application of Knudsen thermal force for detection of inert gases. Results Phys..

[8] Vo D.D., Moradi R., Gerdroodbary M.B., Ganji D.D. (2019). Measurement of low-pressure Knudsen force with deflection approximation for gas detection. Results Phys..

[9] Pikus A., Sebastião I.B., Strongrich A., Alexeenko A. (2019). Characterization of a Knudsen force based vacuum sensor for N2H2O gas mixtures. Vacuum.

[10] Strongrich A., O’Neill W., Cofer A., Alexeenko A. (2014). Experimental measurements and numerical simulations of the Knudsen force on a non-uniformly heated beam. Vaccuum.

[11] Otic C.J.C., Yonemura S. (2022). Mechanism of tangential Knudsen force at different Knudsen numbers. Phys. Fluids.

[12] Li Q., Liang T., Ye W. (2013). Shape-dependent orientation of thermophoretic forces in microsystems. Phys. Rev. E.

[13] Li Q., Liang T., Ye W. (2014). Knudsen torque: A rotational mechanism driven by thermal force. Phys. Rev. E.

[14] Otic C.J.C., Yonemura S. (2022). Effect of different surface microstructures in the thermally induced self-propulsion phenomenon. Micromachines.

[15] Nabeth J., Chigullapalli S., Alexeenko A. (2011). Quantifying the Knudsen force on heated microbeams: A compact model and direct comparison with measurements. Phys. Rev. E.

[16] Zhu T., Ye W. (2010). Origin of Knudsen forces on heated microbeams. Phys. Rev. E.

[17] Zhu T., Ye W., Zhang J. (2011). Negative Knudsen force on heated microbeams. Phys. Rev. E.

[18] Passian A., Warmack R.J., Ferrel T.L., Thundat T. (2003). Thermal transpiration at the microscale: A Crookes cantilever. Phys. Rev. Lett..

[19] Wang X., Zhang Z., Zhang W., Su T., Zhang S. (2020). Impact of improved design on Knudsen force for micro gas sensor. Micromachines.

[20] Ashkin A., Dziedzic J.M., Bjorkholm J.E., Chu S. (1986). Observation of a single-beam gradient force optical trap for dielectric particles. Opt. Lett..

[21] Curtis J.E., Koss B.A., Grier D.G. (2002). Dynamic holographic optical tweezers. Opt. Commun..

[22] Grier D.G. (2003). A revolution in optical manipulation. Nature.

[23] Kessler J.O. (1985). Hydrodynamic focusing of motile algal cells. Nature.

[24] Lee G.B., Hwei B.H., Huang G.R. (2001). Micromachined pre-focused M x N flow switches for continuous multi-sample injection. J. Micromech. Microeng..

[25] Sundararajan N., Pio M.S., Lee L.P., Berlin A.A. (2004). Three-dimensional hydrodynamic focusing in polydimethylsiloxane (PDMS) microchannels. J. Microelectromech. Syst..

[26] Shenoy A., Rao C.V., Schroeder C.M. (2016). Stokes trap for multiplexed particle manipulation and assembly using fluidics. Proc. Natl. Acad. Sci. USA.

[27] Būtaitė U.G., Gibson G.M., Ho Y.L.D., Taverne M., Taylor J.M., Phillips D.B. (2019). Indirect optical trapping using light driven micro-rotors for reconfigurable hydrodynamic manipulation. Nat. Commun..

[28] Setoura K., Tsuji T., Ito S., Kawano S., Miyasaka H. (2019). Opto-thermophoretic separation and trapping of plasmonic nanoparticles. Nanoscale.

[29] Tsuji T., Matsumoto Y., Kugimiya R., Doi K., Kawano S. (2019). Separation of nano- and microparticle flows using thermophoresis in branched microfluidic channels. Micromachines.

[30] Ashkin A., Dziedzic J.M. (1971). Optical levitation by radiation pressure. Appl. Phys. Lett..

[31] Neuman K.C., Block S.M. (2004). Optical trapping. Rev. Sci. Instrum..

[32] Nordine P.C., Atkins R.M. (1982). Aerodynamic levitation of laser-heated solids in gas jets. Rev. Sci. Instrum..

[33] Pan Y.L., Wang C., Hill S.C., Coleman M., Beresnev L.A., Santarpia J.L. (2014). Trapping of individual airborne absorbing particles using a counterflow nozzle and photophoretic trap for continuous sampling and analysis. Appl. Phys. Lett..

[34] Vincenti W., Kruger C. (1965). Introduction to Physical Gas Dynamics.

[35] National Astronomical Observatory of Japan (2006). Rika Nenpyo (Chronological Scientific Tables 2007).

[36] Kaye G., Laby T. (1986). Tables of Physical and Chemical Constants.

[37] Bird G.A. (1994). Molecular Gas Dynamics and the Direct Simulation of Gas Flows.

[38] Nanbu K. (1980). Direct simulation scheme derived from the Boltzmann equation I. Monocomponent gases. J. Phys. Soc. Jpn..

[39] Nanbu K. (1992). Stochastic solution method of the Boltzmann equation I. Mem. Inst. Fluid. Sci..

[40] Nanbu K. (2000). Probability theory of electron–molecule, ion–molecule, molecule–molecule, and Coulomb collisions for particle modeling of materials processing plasmas and cases. IEEE T. Plasma Sci..

[41] Sone Y. (2007). Molecular Gas Dynamics: Theory, Techniques, and Applications.

[42] Sugimoto H., Sone Y. (2005). Vacuum pump without a moving part driven by thermal edge flow. AIP Conf. Proc..

